# Prolapsus Vaginæ in a Filly

**Published:** 1896-08

**Authors:** A. Van Heusden


PROLAPSUS VAGINÆ IN A FILLY. By A. Van Heusden On the 22d of October, 1895, my services were called to a filly 1½ years old. She had been bought at a sale about two weeks before, and then placed in a pasture in the neighborhood of Nimeguen. Shortly before it left the horse-market the attention of the new owner was called to a swelling about the size of a closed fist, which appeared now and then outside of the vulva. The owner thinking it was of no importance put the animal out to pasture. On the 22d of October the owner noticed that the swelling had become much larger, and that the filly strained very much; for this reason he called in my assistance. When I visited the patient I found a dark-reddish black mass outside of the vulva; the animal was straining severely, her respiration was frequent, the pulse increased, and she showed colicky symptoms. On investigation it became clear to me that it was a case of prolapsus of the vagina, and the intestines were present in the prolapsed parts. On pressure the part became the size of a man's head, a part of the rectal mucous membrane also presenting. With gentle pressure the vagina was replaced, but reappeared again immediately afterward. The wall of the vagina was deeply torn at several places, so that I expected the intestines would present every minute. On further investigation it appeared that almost the whole vagina was involved. On the upper wall I could get forward about four cm., but was stopped then by the prolapsed parts. From under it was possible to press even back of the opening of the urethra. In view of the fact that a large part of the wall of the vagina was already necrotic, I gave a very unfavorable prognosis, and the owner concluded to have the animal killed. On post-mortem examination I found that in addition to the severe necrosis the lower wall of the vagina had become abnormally thick. Both horns of the uterus were perfectly normal, so that through the infection the transition from the vagina into the uterus could be more clearly perceived than elsewhere. In my opinion there was first a case of chronic partial prolapsus of the vagina, which suddenly became acute and developed necrosis. How long the prolapsus had complete existence is difficult to say. The former owner probably sold the animal on account of the defect. The present case seemed to me of importance, because in the literature on the subject so far as it is accessible to me only two cases of prolapsus vaginæ in the case of young mares which have never borne colts have been mentioned: One by Koepke in the *Berlin. Archiv*, 1891, and one by Münch in the *Wochenschrift von Adum*, 1891. Koepke saw a case of prolapsus in a three-year-old mare, which was about the size of a man's fist, and which presented suddenly and then disappeared of itself. Münch describes a chronic prolapsus in a four-year-old mare which had never been pregnant. The occurrence of the trouble in such a young mare as the one mentioned is, as far as I know, without a parallel.—*Tijdschrift voor Veeartsenijkunde*, January, 1896.

